# Positive Turn in Elder-Care Workers’ Views Toward Telecare Robots

**DOI:** 10.1007/s12369-021-00841-2

**Published:** 2021-12-02

**Authors:** Tuuli Turja, Sakari Taipale, Marketta Niemelä, Tomi Oinas

**Affiliations:** 1 Faculty of Social Sciences, Tampere University, Kalevantie 5, 33014 Tampere, Finland; 2 University of Jyväskylä, Jyväskylä, Finland; 3 VTT Technical Research Centre of Finland, Tampere, Finland

**Keywords:** Care robots, Ethics, Nurse, Robot acceptance, Values

## Abstract

Robots have been slowly but steadily introduced to welfare sectors. Our previous observations based on a large-scale survey study on Finnish elder-care workers in 2016 showed that while robots were perceived to be useful in certain telecare tasks, using robots may also prove to be incompatible with the care workers’ personal values. The current study presents the second wave of the survey data from 2020, with the same respondents (N = 190), and shows how these views have changed for the positive, including higher expectations of telecare robotization and decreased concerns over care robots’ compatibility with personal values. In a longitudinal analysis (Phase 1), the positive change in views toward telecare robots was found to be influenced by the care robots’ higher value compatibility. In an additional cross-sectional analysis (Phase 2), focusing on the factors underlying personal values, care robots’ value compatibility was associated with social norms toward care robots, the threat of technological unemployment, and COVID-19 stress. The significance of social norms in robot acceptance came down to more universal ethical standards of care work rather than shared norms in the workplace. COVID-19 stress did not explain the temporal changes in views about robot use in care but had a role in assessments of the compatibility between personal values and care robot use. In conclusion, for care workers to see potential in care robots, the new technology must support ethical standards of care work, such as respectfulness, compassion, and trustworthiness of the nurse–patient interaction. In robotizing care work, personal values are significant predictors of the task values.

## Introduction

During the COVID-19 pandemic, the interest in and need for telecare solutions has increased due to physical distancing. Robotic solutions used in home care for older people range from stationary medicine-dispensing robots to robotized locomotion aids [1, 57, 58, 65]. However, it is far from straightforward to robotize any practices in human-centered services. Care work includes fundamental values, tacit knowledge, and interaction between the care worker and the care recipient that are difficult to incorporate in robots. While introducing robots to the care sector, it should be acknowledged that the skills that develop through professional and human(s) care practices ensure that care values are met [67]. Indeed, robots are better suited for assistive or instrumental tasks as opposed to more autonomous or social roles in care practices [47, 52, 61].

Either connected to care work values or independent of them, elder-care workers have questioned the appropriateness of robot use in their work [8, 59, 62]. This has also been the premise in van Wynsberghe’s theory of value-sensitive care robot design, which states that robots should be designed to support and promote the fundamental values of care, for example, patient safety, dignity, and well-being [67]. While there are a considerable number of qualitative studies discussing the theory of value-sensitive care robot design [11, 31, 53], quantitative empirical research on care workers’ value-based views of care robots is very limited. Prior attempts to find value-based explanations for relatively poor robot acceptance among nurses have implied that personal values play a part in care robots’ perceived usefulness [63]. In their cross-sectional study design, Turja et al. [63] showed that compatibility between care robot use and personal values correlates positively with the perceived usefulness of care robots and social norms assigned to them. However, research has left open questions related to causality and the substance of personal values. It is unclear whether changes in value-based views would explain changes in robot acceptance. Moreover, it remains to be studied if social norms or occupational standards underlie the perceived compatibility between robot use and personal values.

The aim of this article was twofold. In the first phase, we utilized longitudinal survey data to see how Finnish elder-care workers’ views toward care robots have changed in the past years as care robots have been gradually introduced to welfare services. We also analyzed what explains the changes in the perceived usefulness of robots in telecare:

- **RQ1** Do elder-care workers express temporal change in the perceived usefulness of telecare robots or perceived personal value–robot use compatibility?

We hypothesized that a positive change in perceived usefulness of telecare robots would be explained by increased experiences with care robots, changed views of value compatibility, and stress caused by COVID-19.

In a second phase focusing on the follow-up data only, we used a cross-sectional study design to look into factors that would explain variations in the value-based assessment of robot use:

- **RQ2** Which factors underlie the perception of personal value–robot use compatibility?

We hypothesized that personal value–robot use compatibility would be associated with social norms toward care robots, work’s meaningfulness, fear of technological unemployment, and COVID-19 stress.

The rest of the article is organized as follows. First, we will introduce the background and the context of this research, including the perceived usefulness of robots, personal values in robot acceptance, and the current use of robots in elder care in Finland. The testing of the hypotheses from the first and the second phases of analysis is reported in Results section and discussed in the final sections of the article.

## Background

Technology acceptance models (TAMs) initially developed by Fred Davis [12], are widely used in research to understand mechanisms behind individuals’ use of and intention to use new technology. Drawing upon the theory of reasoned action [2], TAMs explain technology acceptance by social and functional beliefs, such as perceived usefulness, concerning a certain technology [28]. However, other theoretical traditions have also explained the willingness to adopt new technologies. Venkatesh et al. [66] combined the theory of reasoned action and versions of TAM to several other research models, such as the innovation diffusion theory [51], to create a unified theory of the acceptance and use of technology (UTAUT). As opposed to the TAMs and their focus on the acceptance of particular types of technology, UTAUT models user intentions, performance expectancies, effort expectancies, social influence, and facilitating conditions of technology use in general [20].

In TAMs, the perceived usefulness of technology refers to how using a particular technological solution is seen to enhance an individual’s job performance [12]. In this study, perceived usefulness refers to the instrumental *task value* [29] of a care robot—the possibilities of robot technology to enhance care workers’ job performance. The perceived usefulness of telecare robots is understood as an evaluation of the possible futures and the robot’s role in it.

### Contextual factors predicting robot acceptance

To date, temporal changes in technology acceptance have been mainly associated with the extent of cumulative usage of technology. Consonant with the theory of reasoned action [2], experience and training affect the perceived usefulness of technology positively. Through increased awareness and technology skills, prior experiences also affect one’s views about the future potential of technology [29]. This is of particular importance because the perceived usefulness of an emerging technology, such as service robots, also relies on users’ counterfactual imagination [55]. In other words, without an extensive user experience, people build their expectations toward robots on the little experience they have. Considering these perspectives, there is convincing evidence that habituation and firsthand experiences with technology increase the acceptance of new technology [26, 32]. Hence, it is plausible that the more familiar elder-care workers are with certain types of care robots, the more positive views they have toward robot use in telecare.

- **H1** More extensive experiences with care robots explain the positive turn in the perceived usefulness of robots in telecare.

The extent of prior care robot use can be regarded as a contextual factor. That is, robot usage does not only depend on individual motivation for use, but rather on the availability of robots in the workplace. Another factor we have similarly located between the contextual and the individual is stress. In this study, we focus on the additional strain caused by COVID-19, which could impact the perceived usefulness of robots in telecare. While the pandemic has influenced the functionality of the whole care sector on a structural level, it has caused stress among care workers on the individual level.

The challenge of maintaining good quality and a sufficient amount of care amidst physical distancing, quarantine, isolation orders, and scarce care work resources could be addressed with new remote technologies and robots. In fact, some earlier studies hinted that new technologies may improve well-being and security at work in uncertain and stressful times [22]. COVID-19 has already turned out to be a significant driver of, for example, telecare development [18]. Compared to mere software systems such as video calls on a computer, embodied telecare robots would enable a more pleasant and even committed interaction [35]. Regarding this knowledge, we hypothesize that care workers view the situation with the COVID-19 as emphasizing the need for new telecare technology.

- **H2** COVID-19 stress is associated with more positive perceptions of the usefulness of robots in telecare.

### Compatibility between personal values and using robots at work

Besides COVID-19, economic constraints and various changes in work have increased care workers’ stress [64]. Ethical strain is a form of occupational stress that originates from a conflict between what is thought to be right and what is actually carried out at work [10]. It stems from a situation in which expected or realized actions are incompatible with personal values. Ethical strain can be caused by an organizational culture that is perceived as (at least partially) unethical or by task-related demands that contradict personal values [9, 36].

As a psychological construct of one’s worldview and thoughts on what is right or wrong, personal values play a significant role in human decision-making and attitudes [23]. Hence, compatible values are understood as important motivators for forming opinions and accepting changes. An employee forming an attitude or making a decision on a work-related change evaluates how that change is compatible with or contradictory to their values [27]. For example, some individuals may find that care robotization shows great promise to improve the quality of care in a value-compatible way while others may be prone to view robots as ultimately diminishing human interaction, causing a role conflict between nurses’ values and what is expected of them in a suddenly technologized work environment [4, 13, 56].

The ethical climate in a work environment represents the shared perceptions of practices related to ethical norms and decision-making [64]. The ideal is for the ethical climate to support individual workers’ personal values, because people need to feel and express themselves as competent and moral actors in all their life domains [36]. If people are forced to do work they perceive as unethical, there is a risk of not only ethical stress but also negative emotions, such as self-blame [36]. In rejecting the idea of using robots in care if perceived as contradicting one’s own values, people try to actively prevent role conflict that would result in using working methods that are perceived as personally inappropriate [4].

In a recent study based on a cross-sectional design, Turja et al. [63] suggested that perceived usefulness mediates the relationship between compatibility with personal values and the intention to use a care robot. The compatibility between personal values and technology use originates from the work of Elena Karahanna, who developed the TAM in a more complex and value-based direction. Karahanna et al. [28] broadened the concept of compatible technology to cover four dimensions: compatibility with preferred work style, work practices, prior experiences, and values. Adapting the value-based robot acceptance theory to care robots, there is reason to believe, that robots that are consistent with one’s personal values are likely to be perceived as fostering such values and thereby also supporting the instrumental task value associated with the robotic technology. The assumption, hence, is that perceived personal value–robot use compatibility positively influences beliefs about the usefulness of robotic technology. With reference to Karahanna et al. [28], we hypothesize the following:

- **H3** Increased personal value–robot use compatibility explains the positive turn in the perceived usefulness of robots in telecare.

Social norms provide a theoretical window to further explain value-based compatibility in care robot use. Social norms can contradict robot usage, especially in a human-centered line of work. Although previous studies have acknowledged the dynamic association between personal values and social norms [51], social norms are typically addressed in a one-dimensional way. Following the tradition of TAMs, social norms are usually operationalized as the subjective norm, as in perceived views of “the important others,” for example, coworkers [63]. The social norm toward robots in the workplace manifests itself as either more positive or negative discussions about new technology and robotization among colleagues.

However, in the context of care work, it is essential to take into account another dimension of social norms: the ethical standards of nursing work. The occupational ethical standards of nursing include respectfulness, compassion, partnership, trustworthiness, competence, and safety [45], consistently emphasizing the relationship between the cared for and the carer.

From the perspective of care ethics, empathetic and sympathetic interaction is essential to good care [25]. Care work is relational in the sense that the particularities of each cared-for individual are taken into account in a caring relationship. Care is mainly provided in dyadic relationships that manifest in embodied gestures addressing vulnerability. Moreover, care involves ethical devotion—unselfish attentiveness to another’s vulnerability. Related to devotion, care work has been historically associated with an idea of intrinsic calling, yet contemporary research highlights care work as a vocational occupation [15, 49]. From these ethical perspectives, care work is often considered as meaningful work and the thought of using robots may decrease the feeling of meaningfulness when using new technology in patient-nurse interaction appears to be outside the core of the human-centered work [30].

- **H4** A more positive social norm toward robots in the workplace is associated with higher individual personal value–robot use compatibility.
- **H5** The higher compatibility between occupational ethics and robot use, the higher the personal value–robot use compatibility.
- **H6** Elder-care workers who perceive their work as more meaningful report lower personal value–robot use compatibility.

Besides the perceived meaningfulness of work, merely sustaining a job or career can underlie the perceived (lower) personal value–robot use compatibility. The fear of technological unemployment refers to the threat of losing a job, working hours, or income due to technical progress through which machines replace human labor. Care workers are no exception when it comes to being aware of public discussions about robots taking jobs from people and viewing that as a threat in their own field of work [43]. Hence, we hypothesize that the perception of technological unemployment caused by robots is related to value compatibility of care robot use.

- **H7** Fear of technological unemployment is associated with lower personal value–robot use compatibility.

Furthermore, COVID-19 has emphasized the need to arrange more extensive telecare practices [6]. This acknowledged need may be reflected in more positive views toward robot use among care workers. The pandemic has challenged care work in many ways, the lack of resources being one important aspect of the new situation. In social work, lack of resources is a major factor in ethical strain [40]. However, even if telecare has raised ethical concerns among nurses before [14], we assume that, if anything, the pandemic has reduced these concerns. COVID-19 forces care workers to prioritize safety before physical closeness. We hypothesize that elder-care workers who have felt considerably more strain in their work because of the pandemic stand out with respect to the higher value compatibility of care robot use.

- **H8** COVID-19 stress is associated with higher personal value–robot use compatibility.

### Robots in Elder Care in Finland

Applying robots in welfare services in Finland has been on the governmental agenda since 2016. A particular boosting and networking program, “HyteAiro,” was launched in 2018 to advance the development and use of robots and artificial intelligence in the well-being and health sector [17]. Robots have been mentioned in major strategies to develop care services for older people [38, 39]. Research organizations have produced a roadmap and policy recommendations to develop the Finnish ecosystem and use of care robots in a responsible way [42].

In practice, it seems that other technologies and digital services are being adopted more quickly and widely in care work than robots. For instance, remote home care visits using specific secure video-communication services as well as automated medicine-dispensing services have become more common, particularly during the COVID-19 pandemic [42]. These are paving the way for more developed technology and robotic applications: for instance, medicine-dispensing services can have a robotic physical platform to assist the user in taking medicine independently at home [1].

The maturity of technology limits the implementations of robots in care work. With regard to mobile robots, there have been more single or limited implementations, pilots, or experiments, which often take place in institutional settings. Logistic robot systems have been used in hospitals [34], and small social robots have been adopted for therapy, exercise, and entertainment (e.g., [37, 43]). In rehabilitation institutions, there are both fixed and wearable robots in physiotherapeutic use. Telepresence robots in particular have been experimented with in care facilities and home settings for resident–family communication [44]. Overall, the introduction of robots in care work has been relatively slow, and the care-robot innovation and business ecosystems are still both in their initial steps in Finland [33, 48].

As a future outlook, teleoperation or remote control of robots would solve some of the current maturity problems with more autonomous robots. Teleoperated robots are controlled and monitored by human operators, which makes use safer and the development simpler and more cost-efficient compared to robots that would be autonomous and intelligent enough to make independent observations and decisions. Telepresence robots are the most feasible applications of teleoperated robots used as telecare robots [57]. Telepresence robots typically provide two-way communication between two persons [44]. The robot provides the remote connection with a video-mediated medium, and the remote user can control the movement and functions of the robot in the local space. In addition to mere telecommunication, telecare robots have been developed to provide telerehabilitation [58], daily assistance with tasks such as picking up objects [64], and human–robot interaction [46].

However, new technology must be accepted by users to be adopted into everyday care. Because teleoperated robots would be first and foremost considered tools for care workers, this study investigates how nurses and other professionals view deploying robots in telecare.

## Methods

This longitudinal panel study of care workers’ views toward care robots is based on online survey data from November–December of 2016 (T1) and 2020 (T2). In T1, participants were randomly sampled from the member registers of The Finnish Union of Practical Nurses and The Union of Health and Social Care Professionals in Finland [63]. The respondents were nurses and physiotherapists who worked in elder care services (N = 3800). Of all respondents, 71% reported that their work involved working with patients with dementia.

In T2, the survey was repeated with the same 426 participants from T1 who had expressed their willingness to take part in a follow-up study and were reached via email. The response rate in T2 was 56% (238 respondents). According to the dropout analysis, there were no significant differences in distributions of age, gender, and managerial status between the survey waves. In bivariate mean comparison analysis, there was no indication of selection bias, where more positive views of telecare robots in T1 would have resulted in higher participation in T2.

The majority of the respondents (N = 238) were female (94%) and aged from 24 to 67 at the time of T2 (M = 50.50, SD = 11.30). In terms of occupations, the most common groups were practical nurses (54%) and registered nurses (27%), followed by head nurses (3%) and physiotherapists (2%). The rest (14%) were, e.g., counselors and administrative workers. Almost one-fourth of the respondents had managerial experience (24%). Nearly all (90%) of the practical nurses had a college degree. All registered nurses and physiotherapists had an education from a university of applied sciences. In the residual category of occupations, 43% had a university degree, 53% a college degree, and 4% had lower levels of education.

In the data from 2020, most of the respondents did not have any experience with care robots (57%), while almost a third (30%) reported having experience with one type of care robot (8% having experience with two robot types and 5% with three or more). From the robot types listed, 24% of the respondents were familiar with a medicine-dispensing robot typically used in home care and 14% with a robotic pet, such as Paro. Less familiar robot types were physiotherapy robots (familiar to 7%), patient-lifting robots (familiar to 6%), and telepresence, logistic, and social robots (e.g., Nao), which were all familiar to 5% of the respondents. Robot experience was tested as an independent variable in Phase 1 and as a control variable in Phase 2.

When answering the questionnaire, respondents were guided by robot definitions in writing and pictures. The definition of a robot was borrowed from a Eurobarometer questionnaire: “A robot is defined as a machine which can assist humans in everyday tasks without constant guidance or instruction, e.g., as a kind of coworker helping on the factory floor or as a robot cleaner, or in activities which may be dangerous for humans, like search and rescue in disasters. Robots can come in many shapes or sizes and some may be of human appearance” [16]. Before the questions about telecare robots, it was additionally explained how robots can be either human-operated or more autonomous.

The study complies with the regulations of the Finnish Advisory Board of Research Integrity and more broadly with the Declaration of Helsinki. All of the participants were informed about the aims of the study and they had the right to decline participation. Consent was requested at the beginning of the survey and the data handling was designed to ensure the participants' anonymity.

### Variables

The descriptive information for the variables used in the analysis is drawn from the longitudinal data used (N = 238).

**Fig. 1:**
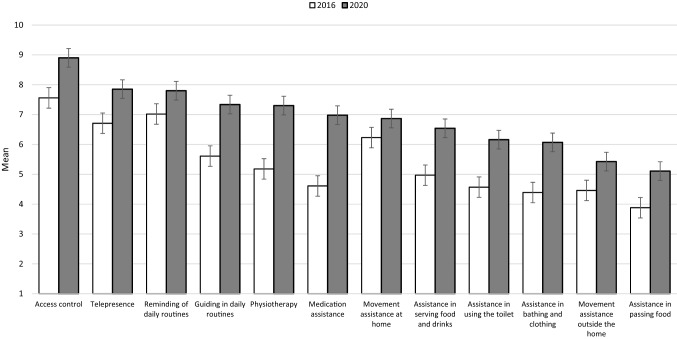
Perceived usefulness of telecare robots, means per scenario, per year

*Perceived usefulness of telecare robots* was measured by twelve items presenting robotic assistance in different scenarios: “How would you consider telecare robots’ usefulness in different tasks? In your answers, orient to the elder care services you are currently working in.” This part of the questionnaire was identical between T1 and T2. The respondents evaluated the usefulness of robotic assistance in each scenario on a scale from 1 to 10. As illustrated in Fig. 1, there was a systematic change from lower to higher perceived usefulness in each scenario. There were also changes in the internal order of what was considered least and most useful, on average. For example, the idea to use robots in medication assistance climbed in rank from 8 to 6. For the multivariate analysis, an aggregate mean-of-means variable summing up the twelve items was constructed for both T1 (M = 5.31, SD = 2.27, α = 0.95) and T2 (M = 6.84, SD = 1.97, α = 0.91).

As a novel research topic, perceived usefulness of telecare robots measure was self-developed in 2016. The items of perceived usefulness, along with the rest of the questionnaire, were developed and piloted together with care professionals: first in a focus group discussion of five persons, and later with other 13 care professionals who filled out an online version of the questionnaire. The reason for the two-step development of the survey questionnaire was to test and improve its face validity, relevance and professional appropriateness.

*The perceived personal value–robot use compatibility* was measured by three items, identical between T1 and T2. The statements were modified from the information system acceptance questionnaire validated by Karahanna et al. [28]: 1) “Using care robots runs counter to my own values”; 2) “Using care robots does not fit the way I view the world”; and 3) “Using care robots is not appropriate for a person with my values when thinking about the role of robots.” The statements were translated into Finnish by professionals. The response scales ranged from totally agree to totally disagree, where higher values indicated incompatibility. An aggregate variable was formed for T1 (M = 3.40, SD = 1.15, α = 0.93) and for T2 (M = 2.33, SD = 1.08; α = 0.88). For the final analysis, the scale was reversed for a more intuitive interpretation of value-compatibility. When used as a dependent variable in the second phase of analysis, the non-normally distributed personal value–robot use compatibility of T2 was dichotomized (Md = 2) into indication for value incompatibility (0) *versus* value compatibility (1).

*Social norms toward care robots* were operationalized as a dual explanatory factor in T2. Using a response scale from totally agree to totally disagree, the first statement was about the subjective norm in the workplace: “My colleagues have mainly a welcoming attitude toward care robots” (M = 2.97, SD = 0.87), while the second one measured occupational ethics–robot use compatibility: “The norms and standards in my work would be a challenge in ethical use of care robots” (M = 2.71, SD = 1.16). These two items were treated as separate factors, with, after standardizing the scales, higher values indicating more accepting norms concerning robots.

*Perceived meaningfulness of work* was measured in T2 with five items and a Likert scale from 1 to 4: (1) I perceive my work as meaningful; (2) I feel that my work is important; (3) I know that my work has positive effect in the world; (4) I have found work that has meaningful objectives; (5) My work has impact to some larger goal. The aggregate variable, in which higher values indicate the meaninglessness of work, shows a relatively high perceived meaningfulness of work among the sample (M = 1.69, SD = 0.59, α = 0.85).

*Fear of technological unemployment* was measured in T2 by a single item because a planned double-item construct turned out to be internally inconsistent. The statement “I fear that robots will replace or reduce my work” had a response scale from 1 to 4 (M = 3.45, SD = 0.69) and ended up in the analysis as a significant explanatory factor. The statement about delegating tasks to a robot in terms of threatening one’s livelihood did not prove to be a significant factor in the analysis and was thus excluded from final analyses.

*COVID-19 stress* was measured in T2 by a single item. The statement “COVID-19 has increased work stress beyond my resources” had a response scale from 1 to 4 in which higher values indicated a higher level of perceived stress (M = 2.06, SD = 0.89).

### Statistical Analysis

In the first phase of this study, the objective was to investigate the change in views toward care robots and the effects of that change. We conducted ANOVA for repeated measures and fixed effects (FE) within-regression analysis with a post-hoc FE model. We employed the FE approach, which models changes within individuals over time and controls all time-constant individual characteristics for its strength compared to, for example, a random effects (RE) approach. FE controls the effects of any observed or unobserved time-invariant personal characteristics of respondents [3]. By employing within-individual variation in estimation, the FE approach makes any causal claims more warranted compared to cross-sectional correlations [68]. In terms of causal modelling, in FE estimation individuals serve as their own controls—that is, we are comparing how change in independent variables is related to change in dependent variables. In the second phase, we used binary logistic regression analysis in the cross-sectional study design of T2.

## Results

To learn about the potential relationships among the attitudes toward telecare robots and the compatibility between personal values and robot-use in two measuring points, pairwise correlations were computed (Table 1). While scores within T1 and T2 were strongly correlated, the scores between measuring points were not. This was the first indication of a significant temporal change among individuals and their views on care robots.

**Table 1:** Correlation matrix

			1	2	3	4	5	6	7	8
1	Usefulness of telecare robots T1	*r*	1							
		*N*	156							
2	Usefulness of telecare robots T2	*r*	− 0.02	1						
		*N*	155	211						
3	Personal value–robot use compatibility T1	*r*	0.59**	0.01	1					
		*N*	156	188	190					
4	Personal value–robot use compatibility T2	*r*	0.08	0.46**	0.06	1				
		*N*	156	211	190	213				
5	Occupational ethics-robot use compatibility T2	*r*	0.14	0.27**	0.15*	0.46**	1			
		*N*	155	210	189	212	212			
6	COVID-19 stress T2	*r*	0.06	− 0.09	0.04	− 0.06	− 0.01	1		
		*N*	134	186	166	188	188	188		
7	Fear of technological unemployment T2	*r*	− 0.05	0.10	0.10	0.28**	0.07	− 0.27^**^	1	
		*N*	130	179	160	181	181	179	181	
8	Subjective norm in the workplace T2	*r*	0.00	0.21**	0.01	0.26**	0.15*	− 0.01	0.01	1
		*N*	137	191	170	193	192	188	181	193
9	Meaningfulness of work T2	*r*	0.01	0.01	− 0.03	− 0.12	0.10	− 0.21**	0.14	0.09
		*N*	135	188	168	190	190	187	180	190

**Correlation is significant at the 0.01 level (2-tailed)

*Correlation is significant at the 0.05 level (2-tailed)

Pearson correlation analysis also showed that if colleagues perceived the use of care robots as acceptable (subjective norm) and meeting the ethical standards of care work (compatibility with occupational ethics), the respondents were more prone to have higher expectations toward the usefulness of telecare robots as well as higher perception of compatibility between personal values and robot use. Fear of technological unemployment caused by robots had a negative correlation with COVID-19 stress, indicating that respondents who reported COVID-related stress were on the average less concerned about losing work to robots.

In repeated measures using ANOVA, we found that expectations regarding the usefulness of telecare robots had increased considerably in time, comparing the average scores of T1 (M = 5.31, SD = 2.27) and T2 (M = 6.84, SD = 1.97; (F(1) = 40.33, *p* < 0.001, ηp^2^ = 0.21). The differences approximately followed normal distribution.

Concerns regarding the compatibility between the use of care robots and personal values had also decreased from T1 (M = 3.40, SD = 1.15) to T2 (M = 2.33, SD = 1.08; (T = 2590, *p* < 0.001, d = 0.68). This difference was notable considering that the mode in the perceived compatibility between personal values and robot use changed from “somewhat incompatible” (T1) to “totally compatible” (T2).

These results were consistent and independent of the respondents’ occupation and education level or how much firsthand experience they had with care robots. This was despite the fact that the respondents’ familiarity with care robots had doubled over two years (T1: 21% vs. T2: 43%).

Testing the Phase 1 hypotheses (H1–H3), the results showed how the temporal change in views about telecare robots had a significant interaction with personal value–robot use compatibility (F[1] = 10.21, *p* < 0.005, ηp^2^ = 0.06), but not with increased experience with care robots or the stress caused by COVID-19.

### Within-Estimations of Robot Usefulness in Telecare

In order to analyze the effect of value-based assessment on the perceived usefulness of robots in telecare, we used fixed effect analysis. Results show how a change in the compatibility between personal values and robot use has caused a change in the perceived usefulness of telecare robots (Table 2). Fixed effects analysis was rationalized for its ability to reduce the impact of confounding by time-invariant and unmeasured individual-level factors [21].

**Table 2:** Fixed effects: Perceived usefulness of telecare robots

Constant	2.52***
Personal value–robot use compatibility	0.38*
Time	1.76***
Number of groups	232
Within-subject SD	1.76
Between-subject SD	2.10
Within-subject R^2^	0.24
Between-subject R^2^	0.01
Overall R^2^	0.10

**Correlation is significant at the 0.01 level (2-tailed)

*Correlation is significant at the 0.05 level (2-tailed)

Although a majority of the respondents had developed more positive views (difference ranging from 0 to 4) of care robots in time, one fifth of the respondents showed an opposite, negative trend (difference ranging from − 3 to 0) in compatibility between personal values and robot use. Because of the asymmetric effects, we conducted post-hoc tests separately for positive and negative temporal effects. As a result, the change in the perceived usefulness of telecare robots was predicted more by the effect where respondents had an increased, rather than decreased, perception of the compatibility between personal values and robot use. However, the different-direction effects in this compatibility did not prove to differ significantly from each other (F[1] = 1.39, *p* = 0.24).

### Cross-Sectional Analysis of Value-Based Attitudes

To examine the nature of personal values more closely, we conducted a cross-sectional analysis. The Phase 2 hypotheses (H4–H8) regarding the compatibility between personal values and robot use were tested in a binary logistic regression model with explanatory factors available in a T2 data set. The results are presented in Table 3. The model showed an excellent goodness of fit (χ^2^(8) = 3.59, *p* = 0.89) with an indicative predictive power of 30–40% of the variation in perceived compatibility between personal values and robot use.

**Table 3:** Binary logistic regression of personal value–robot use compatibility

	OR	95% C.I		p
		Lower	Upper	
Age	1.027	0.997	1.059	0.082
Gender	1.341	0.337	5.338	0.678
Managerial experience	3.141	1.239	7.963	0.016
Robot experience	1.049	0.717	1.535	0.806
Subjective norm in the workplace	1.420	0.929	2.169	0.105
Occupational ethics-robot use compatibility	2.024	1.426	2.872	< 0.001
Meaningfulness of work	7.841	0.721	85.301	0.091
Fear of technological unemployment	0.301	0.158	0.574	< 0.001
COVID-19 stress	4.460	1.157	17.191	0.030
COVID-19 stress * Meaningfulness of work	0.387	0.175	0.855	0.019
Constant	0.003			0.014
Cox&Snell R^2^ = 0.302, Nagelkerke R^2^ = 0.403				

Personal value–robot use compatibility was associated with managerial status, compatibility between occupational ethics and robot use, less fear of technological unemployment and higher COVID-19 stress. These results were consistent over the respondents’ age, gender or firsthand experience with care robots. The perceived social norm assigned to care robots in the workplace did not prove to be a significant factor in compatibility between personal values and robot use. The results imply that social norms behind robot acceptance originated from more universal, ethical standards of care work rather than shared attitudes in the respondent’s workplace. The odds of care workers recognizing the use of care robots as value-compatible were two times higher when they perceived robot use as compatible with occupational ethics.

The fear of technological unemployment was another highly significant factor when it came to the threshold of accepting robot use as value-compatible. The respondents who expected robots to replace care work were prone to perceive robot use as incompatible in care work, and vice versa, those with less fear about robots causing their unemployment had more positive views on average. Furthermore, the elder-care workers who found that COVID-19 has been a major stressor had a higher probability of perceiving care robots as value-compatible rather than incompatible. The work-related demands heightened by the pandemic were perceived as major stressors among the majority of the respondents (69%).

Post-hoc analysis focused on interactions among explanatory factors, and only significant interaction was found between the meaningfulness of work and COVID-19 stress. Although the meaningfulness of work alone did not emerge as a significant factor in personal value–robot use compatibility, the feelings of doing meaningful work combined with higher COVID-19 stress resulted together in a higher probability of personal value–robot use compatibility.

As for the background variables, the respondents with managerial experience, such as head nurses, stood out with the most positive views of personal value–robot use compatibility. The fact that managerial experience almost triples the odds of having no conflict between care robot use and personal values may imply objectives of cost-effectiveness and effective organization of work in robotization as well as a more secure position when facing technological changes. Fear of technological unemployment caused by robots was a separate, significant explanatory factor of personal value–robot use compatibility, but the interaction between managerial status and the fear of technological unemployment did not reach statistical significance in this data.

## Discussion

In this study, we investigated and found a positive turn in robot acceptance among elder-care workers. The Finnish care workers’ views toward robot assistance in telecare had changed in the positive direction between 2016 and 2020. The temporal change was explained by more preferable views of personal value–robot use compatibility. Hence, *personal values* predicted the *task value* of robots.

Table 4 summarizes the hypotheses and the outcomes of hypothesis testing. In the first phase of the analysis, we focused on temporal changes in care workers’ views toward care robots. In the second phase, we focused on the research question regarding the factors underlying personal value–robot use compatibility.

**Table 4:** Tested hypotheses in Phases 1 and 2

Phase of the analysis	Hypothesis		Outcome
Phase 1	H1	More extensive experience with care robots explains the positive turn in the perceived usefulness of robots in telecare	Rejected
	H2	COVID-19 stress is associated with more positive perceptions of the usefulness of robots in telecare	Rejected
	H3	Increased personal value–robot use compatibility explains the positive turn in the perceived usefulness of robots in telecare	Supported
Phase 2	H4	A more positive social norm toward robots in the workplace is associated with higher individual personal value–robot use compatibility	Rejected
	H5	The higher the occupational ethics-robot use compatibility, the higher the personal value–robot use compatibility	Supported
	H6	Elder-care workers who perceive their work as more meaningful report lower personal value–robot use compatibility	Rejected
	H7	Fear of technological unemployment is associated with lower personal value–robot use compatibility	Supported
	H8	COVID-19 stress is associated with higher personal value–robot use compatibility	Supported

### The Positive Change in Views Toward Telecare Robots (Phase 1)

Neither of the partly contextual factors—the increased experience with robots or the strains associated with the worldwide pandemic—affected the perceived task value of telecare robots, and hence, H1 and H2 were rejected. Regarding H1, the lack of support for the relationship between prior experience and more positive views of usefulness could result from the fact that prior experiences were estimated by quantitative means only. The quality of the prior experiences were beyond the reach of this study and we cannot tell if experiences of *positive* nature, for instance, would have associated with higher perceptions of telecare robot usefulness.

The findings support H3 by implying that value-based evaluation has a notable role in the acceptance of care robots. The belief that the use of care robots is compatible with personal values was the only construct to explain the temporally changed views about telecare robots. Care workers have the ability to observe and predict moral dilemmas when it comes to organizational and technological changes. Care robots or telecare robots can only be viewed as useful forms of care work if and when the robots are considered appropriate and value-compatible at a principle level. When robots are introduced to care, workers make value-based evaluations on robots’ prerequisites for being useful or for causing ethical stress and role conflicts [4, 36]. Our findings are in line with studies where the intended usage of robots is decreased when robots are viewed as a threat to human jobs and safety, as well as human identity and uniqueness [24].

Although still about one third of the respondents perceived robots as non-compatible with care work on a principle level, more often than before, the use of care robots was viewed as value-compatible. This positive turn also reflected the more positive views on the usefulness of robots in telecare. As care robots become more familiar, the reality of what robots can and cannot do becomes more evident to potential users. The expectations and fears put into perspective may explain how care robots are seen in a more positive light. There is prior evidence of robotic assistants being viewed more positively if they are considered to be tools and equipment [47, 52, 61]. Hence, a gradual revelation that this is exactly what the robots are capable of in their current stage of development and maturity can well explain the more positive views toward care robots in telecare, as well.

Care robots becoming more familiar without necessarily any increased firsthand user experience, includes also a dimension of a societal-political discourse. Frennert and Baudin [19] studied the perspectives of municipal elder-care actors in Sweden on welfare technology (including robots). They identified a number of reasons why welfare technology is adopted in care at such a slow pace, but they also found that both potential users and decision-makers had positive views about the technology. Two mechanisms were provided to explain this discrepancy, and both may apply to the situation in Finland.

First, Frennert and Baudin [19] suggest that the political discourse surrounding welfare technology influences attitudes. The Swedish government has launched a strategy called “eHealth 2025” that sets a vision and goals for using technology to improve health and welfare services, and the pro-technology arguments may have been learned also among eldercare actors. In Finland, similar agendas and arguments promoting the use of robots are written in the programs and strategies of the government (e.g., [17]). With regard to timing, the positive turn in the Finnish data fits well into the timeline in which the programs and strategies have been published.

The second explanation of Frennert and Baudin [19] is that the successful technology experiences in one place makes attitudes more positive elsewhere. The Swedish government has funded a selected group of municipalities to conduct experiments in integrating welfare technologies into care. These experiments have been reported widely throughout the nation. The experiences and changed practices in the experimenting municipalities may have influenced how the technologies are being perceived in other municipalities. In Finland, the KATI program^1^ is a similarly coordinated activity and initiative for municipal experimentation and dissemination of best practices in the use of technology. Timewise the KATI program will be implemented in 2021, but the initiatives made already in 2020 can play a part in how the views toward telecare robots have changed in a more positive direction. One example of telecare robots that have become more common in Finnish home care are the medication dispensing robots. In our data, elder-care workers perceived medication dispensing robots as highly useful, much more so than in 2016.

### The Factors Underlying Personal Values (Phase 2)

For the distinct nature of care work as a context to be robotized, we further investigated the substance of value-compatibility in the second phase of this study. The threshold of personal value–robot use compatibility was examined by explanatory factors of social norms, the meaningfulness of work, the perceived threat of technological unemployment and COVID-19 stress. As a result, personal value–robot use compatibility was more likely among elder-care workers who felt that care-robot use is aligned with the ethical standards of care work (H5), who had less fear of technological unemployment (H7), and who reported higher COVID-19 stress (H8).

The importance of occupational ethics-robot use compatibility emphasizes how the development and deployment of robots in care should aim at designing and investing in technology that supports the core values of care work. This finding is in line with prior studies of care workers’ value-driven attitudes toward robot use [8, 59]. Value-based assessment of robot acceptance seems to refer mostly to occupational ethics and the fear of technological unemployment, both of which are associated with care work as a human-centered work where dyadic interaction between people—the carer and the cared—is prioritized [25]. The results also bring evidence for how viewing the possibilities to use robots in care work, the universal ethical standards of care work stand out. The use of care robots is accepted as a value-compatible change if it is in line with ethical standards of care work, more so than the social norms in a workplace with a however closer social environment. As the perceived social norms of the workplace did not even reach a significant association with personal value–robot use compatibility, H4 had to be rejected.

Workplace norms seem to have little leverage when it comes to competing with occupational ethical standards of care work, which are considered as something nurses are committed to as individuals and as a community [5]. This commitment beyond any organizational limits is bound to regulate all decision-making in nursing work, whether it is assessing operations, actions, or emerging changes in the occupation [50]. Similar to how the ethical climate is found to mediate the relationship between ethical stress and job satisfaction [64], ethical climate has potential to affect the perceived personal value–robot use compatibility.

Ethical strain at work is considered to be negative, excessive stress, and should be prevented to maintain workers’ motivation, job satisfaction, and commitment [7, 64]. Ethical strain also relates to organizational changes. Sometimes the compatibility between work and personal values is evaluated already when choosing a career. If one chooses a certain field of study or a career based on their expectations for the high ethical standards and social responsibility associated with the line of work [40, 68], it makes organizational changes more complicated. Meaningful, motivating and satisfactory work is something to pursue also during and after technological changes.

In this study, personal value–robot use compatibility was viewed as acceptable among the elder-care workers who reported higher COVID-19 stress, and this association was boosted by feelings of the work’s meaningfulness. Not only was the hypothesis (H6) of the negative association between work’s meaningfulness and personal value–robot use compatibility rejected, but also the results of the interaction implied an opposite effect than what was expected. Those who perceived work as meaningful and at the same burdened by the current pandemic, reported higher personal value–robot use compatibility. This is interpreted as the COVID-19 situation highlighting the demands of elder-care and how a resolution is anticipated through the use of new technology. Contrary to what was expected, to find care work as meaningful is actually to value robot assistance as one way to relieve stress on the job.

The most significant underlying values in personal value–robot use compatibility included both social values in terms of occupational ethics and individual values in terms of fear of unemployment. Whereas occupational ethics are centered on patient wellbeing and hence refer to moral, universal, and benevolent values, care workers’ fear of unemployment refers to instrumental and more self-centered values [54, 60]. Actually, several findings in this study—beyond the direct question about the fear of technological unemployment—supported the important role that trust in maintaining work, even in robotization, has on value-based robot acceptance. Elder-care workers with managerial experience had a high probability of perceived personal value–robot use compatibility which may be partly explained by a more secure job and career. Moreover, those who found care work particularly meaningful in a stressful COVID-19 situation found robot use to be value-compatible, which does not imply any specific fear of technological unemployment.

### Limitations

Although they provide new information about the change in robot acceptance, our results do not indicate whether the change is enduring instead of a periodic change between the two years when the data were collected. In future studies, the longitudinal effect in the perceived usefulness of care robots will be improved through the use of more than two measuring points.

Another limitation is that we were not able to test all the hypotheses in one model of multiple regressions because the sample size in T2 limited the range of analysis. The small sample size of T2 was due to a data collection strategy where only volunteers were invited to participate in the follow-up survey.

Finally, as an obvious limitation, the findings are generalizable to Finnish care workers only. For this reason, cross-cultural studies are highly recommended.

## Conclusion

Robotizing elder-care has raised many ethical and practical concerns in discussions about the nature of human-centered and sensitive service work. In prior theoretical studies it has been assumed that healthcare professionals see a moral dilemma in robotizing care, which can result in rejection of all robot use. This research has brought unique empirical evidence to the matter and has proven the significance that value-based evaluation has in the acceptance of care robots. The temporal change in views toward the usefulness of telecare robots was affected not simply by an increase in experiences with robots, but by higher personal value–robot use compatibility. Care workers felt on average—and more often than before—that the use of care robots is compatible with their personal values.

## Data Availability

Data can be shared only after the project, in 2022.

## Footnotes

1 KATI is a technology supporting smart ageing and care at home program. https://thl.fi/en/web/thlfi-en/research-and-development/research-and-projects/technology-supporting-smart-ageing-and-care-at-home-programme-kati.

## References

[1] Airola E, Rasi P (2020). Domestication of a robotic medication-dispensing service among older people in Finnish Lapland. Hum Technol Interdiscip J Hum ICT Environ.

[2] Ajzen I, Fishbein M (1980). Understanding attitudes and predicting social behavior.

[3] Allison P (2009). Fixed effects regression models.

[4] Beehr TA, Glazer S, Barling J, Kelloway EK, Frone MR (2005). Organizational role stress. Handbook of work stress.

[5] Benjamin M, Curtis J (2010). Ethics in nursing: cases, principles, and reasoning.

[6] Bhaskar S (2020). Designing futuristic telemedicine using artificial intelligence and robotics in the COVID-19 era. Front Public Health.

[7] Borhani F, Abbaszadeh A, Nakhaee N, Roshanzadeh M (2014). The relationship between moral distress, professional stress, and intent to stay in the nursing profession. J Med Ethics Hist Med.

[8] Coco K, Kangasniemi M, Rantanen T (2018). Care personnel's attitudes and fears toward care robots in elderly care: a comparison of data from the care personnel in Finland and Japan. J Nurs Scholarsh.

[9] Colaco B, Loi NM (2019) Investigating the relationship between perception of an organisation’s ethical culture and worker motivation. Int J Organ Anal

[10] Corley MC, Minick P, Elswick RK, Jacobs M (2005). Nurse moral distress and ethical work environment. Nurs Ethics.

[11] Cresswell K, Cunningham-Burley S, Sheikh A (2018). Health care robotics: qualitative exploration of key challenges and future directions. J Med Internet Res.

[12] Davis F (1989). Perceived usefulness, perceived ease of use, and user acceptance of information technology. Manag Inf Syst Q.

[13] Drummond JS, Standish P, Drummond JS, Standish P (2007). Introduction: philosophical enquiry into education. The philosophy of nurse education.

[14] Eccles A (2015). Telecare technologies and isolation: some ethical issues. Smart Homecare Technol TeleHealth.

[15] Emerson C (2017). Calling to nursing. Adv Nurs Sci.

[16] Eurobarometer (2014) Public attitudes towards robots. WWW data archive, Gesis. 10.4232/1.12265

[17] Finnish Institute for Health and Welfare (2021) The Well-being and Health Sector’s Artificial Intelligence and Robotics Programme (Hyteairo). https://thl.fi/en/web/thlfi-en/research-and-development/research-and-projects/the-well-being-and-health-sector-s-artificial-intelligence-and-robotics-programme-hyteairo

[18] Fisk M, Livingstone A, Pit SW (2020). Telehealth in the context of COVID-19: changing perspectives in Australia, the United Kingdom, and the United States. J Med Internet Res.

[19] Frennert S, Baudin K (2019). The concept of welfare technology in Swedish municipal eldercare. Disabil Rehabil.

[20] Ghazali AS, Ham J, Barakova E, Markopoulos P (2020). Persuasive robots acceptance model (PRAM): roles of social responses within the acceptance model of persuasive robots. Int J Soc Robot.

[21] Gunasekara FI, Richardson K, Carter K, Blakely T (2014). Fixed effects analysis of repeated measures data. Int J Epidemiol.

[22] Holland J, Kingston L, McCarthy C, Armstrong E, O’Dwyer P, Merz F, McConnell M (2021). Service robots in the healthcare sector. Robotics.

[23] Homer PM, Kahle LR (1988). A structural equation test of the value-attitude-behavior hierarchy. J Pers Soc Psychol.

[24] Huang HL, Cheng LK, Sun PC (2021). The effects of perceived identity threat and realistic threat on the negative attitudes and usage intentions toward hotel service robots: the moderating effect of the robot’s anthropomorphism. Int J Soc Robot.

[25] Hämäläinen A (2020). Responses to vulnerability: care ethics and the technologisation of eldercare. Int J Care Caring.

[26] Höflich JR, El Bayed A, Vincent J, Taipale S, Sapio B, Lugano G, Fortunati L (2015). Perception, acceptance, and the social construction of robots—exploratory studies. Social robots from a human perspective.

[27] Jonge ED (2014). Profession or craft? A reflection on the moral identity of social work. J Soc Interv Theory Pract.

[28] Karahanna E, Agarwal R, Angst CM (2006). Reconceptualizing compatibility beliefs in technology acceptance research. Manag Inf Syst Q.

[29] Karahanna E, Straub DW (1999). The psychological origins of perceived usefulness and ease-of-use. Inf Manag.

[30] Kilponen K, Huhtala M, Kinnunen U, Mauno S, Feldt T (2021). Illegitimate tasks in health care: illegitimate task types and associations with occupational well-being. J Clin Nurs.

[31] King BM, Barry CD (2019). “Caring between” the nurse, the one nursed, and the healthcare robot: an interpreted nursing situation using the Barry, Gordon, King Framework. Int J Hum Caring.

[32] King WR, He J (2006). A meta-analysis of the technology acceptance model. Inf Manag.

[33] Lanne M, Tuisku O, Melkas H, Niemelä M (2020). My business or not? The perspective of technology companies on shifting towards care robotics. Eur Plan Stud.

[34] Lappalainen I (2019) Logistics robots as an enabler of hospital service system renewal? In: Gummesson E, Mele C, Polese F (eds) The 10 years Naples forum on service. Service dominant logic, network and systems theory and service science: integrating three perspectives for a new service agenda. Ischia, Italy

[35] Matarić MJ, Eriksson J, Feil-Seifer DJ, Winstein CJ (2007). Socially assistive robotics for post-stroke rehabilitation. J Neuro Eng Rehab.

[36] Meier LL, Semmer NK, Spector P (2013) Unethical work behavior as a stressor. In: Handbook of unethical work behavior, pp 168–179

[37] Melkas H, Hennala L, Pekkarinen S, Kyrki V (2020). Impacts of robot implementation on care personnel and clients in elderly-care institutions. Int J Med Inf.

[38] MSAH (2020) Ministry of social affairs and health and association of finnish local and regional authorities. Quality recommendation to guarantee a good quality of life and improved services for older persons 2020–2023. The Aim is an Age-friendly Finland. Publications of the Ministry of Social Affairs and Health 2020:29. http://urn.fi/URN:ISBN:978-952-00-5457-1

[39] MSAH (2020) National programme on ageing 2030. For an age-competent Finland. Publications of the Ministry of Social Affairs and Health 2020:31

[40] http://urn.fi/URN:ISBN:978-952-00-6865-3

[41] Mänttäri-van der Kuip M (2016). Moral distress among social workers: the role of insufficient resources. Int J Soc Welf.

[42] Ng ES, Gossett CW (2013). Career choice in Canadian public service: an exploration of fit with the millennial generation. Public Pers Manag.

[43] Niemelä M, Heikkinen S, Koistinen P, Laakso K, Melkas H, Kyrki V (eds) (2021) Robots and the future of welfare services—a Finnish roadmap. Aalto University publication series CROSSOVER, 4/2021. http://urn.fi/URN:ISBN:978-952-64-0323-6

[44] Niemelä M, Määttä H, Ylikauppila M (2016) Expectations and experiences of adopting robots in elderly care in Finland: perspectives of caregivers and decision-makers. ICServ 2016 special session: meaningful technologies for seniors (6.-8.9.2016). Tokyo, Japan

[45] Niemelä M, Van Aerschot L, Tammela A, Aaltonen I, Lammi H (2019). Towards ethical guidelines of using telepresence robots in residential care. Int J Soc Robot.

[46] NMC (2015) The code. Professional standards of practice and behaviour for nurses and midwives. Nursing and Midwifery Council. https://www.nmc.org.uk/globalassets/sitedocuments/nmc-publications/nmc-code.pdf

[47] Ogawa K, Nishio S, Koda K, Taura K, Minato T, Ishii CT, Ishiguro H (2011) Telenoid: tele-presence android for communication. In: ACM SIGGRAPH 2011 emerging technologies. 2011. Vancouver, British Columbia, Canada: Association for Computing Machinery. 10.1145/2048259.2048274

[48] Parviainen J, Turja T, Van Aerschot L, Korn O (2019). Social robots and human touch in care: the perceived usefulness of robot assistance among healthcare professionals. Social robots. An interdisciplinary compendium on technological, societal and ethical aspects.

[49] Pekkarinen S, Tuisku O, Hennala L, Melkas H (2020). Robotics in Finnish welfare services: dynamics in an emerging innovation ecosystem. Eur Plan Stud.

[50] Raatikainen R (1997). Nursing care as a calling. J Adv Nurs.

[51] Rautava-Nurmi H, Westergård A, Henttonen T, Ojala M (2013) Hoitotyön taidot ja toiminnot [Care work skills and actions] Helsinki, Finland: Sanoma Pro Oy

[52] Rogers EM (1983). Diffusion of innovations.

[53] Savela N, Turja T, Oksanen A (2017). Social acceptance of robots in: different occupational fields: a systematic literature review. Int J Soc Robot.

[54] Schoenhofer SO, van Wynsberghe A, Boykin A (2019). Engaging robots as nursing partners in caring: nursing as caring meets care-centered value-sensitive design. Int J Hum Caring.

[55] Schwartz SH, Hewstone M, Brown RJ (1992). Universals in the content and structure of values. Advances in experimental social psychology.

[56] Seibt J (2021) As if it were a person. Artificial sociality as call for robophilosophy. Tampere philosophy research seminar. 4 Mar 2021

[57] Sellman D (2010). Professional values and nursing. Med Health Care Philos.

[58] Shishehgar M, Kerr D, Blake J (2018). A systematic review of research into how robotic technology can help older people. Smart Health.

[59] Song A, Wu C, Ni D, Li H, Qin H (2016). One-therapist to three-patient telerehabilitation robot system for the upper limb after stroke. Int J Soc Robot.

[60] Suwa S, Tsujimura M, Kodate N, Donnelly S, Kitinoja H, Hallila J, Yu W (2020). Exploring perceptions toward home-care robots for older people in Finland, Ireland, and Japan: a comparative questionnaire study. Arch Gerontol Geriatr.

[61] Toode K, Routasalo P, Helminen M, Suominen T (2015). Hospital nurses’ work motivation. Scand J Caring Sci.

[62] Turja T, Parviainen J (2020) The use of affective care robots calls forth value-based consideration. In: 2020 29th IEEE international conference on robot and human interactive communication (RO-MAN). IEEE, pp 950–955

[63] Turja T, Van Aerschot L, Särkikoski T, Oksanen A (2018). Finnish healthcare professionals’ attitudes toward robots: reflections on a population sample. Nurs Open.

[64] Turja T, Aaltonen I, Taipale S, Oksanen A (2020). Robot acceptance model for care (RAM-care): a principled approach to the intention to use care robots. Inf Manag.

[65] Ulrich C, Odonnell P, Taylor C, Farrar A, Danis M, Grady C (2007). Ethical climate, ethics stress, and the job satisfaction of nurses and social workers in the United States. Soc Sci Med.

[66] Van Osch M, Bera D, Van Hee K, Koks Y, Zeegers H (2014). Tele-operated service robots: ROSE. Autom Constr.

[67] Venkatesh V, Morris MG, Davis GB, Davis FD (2003). User acceptance of information technology: toward a unified view. Manag Inf Syst Q.

[68] van Wynsberghe A (2015). Healthcare robots: ethics, design and implementation.

[69] Wooldridge J (2010). Econometric analysis of cross section and panel data.

[70] Yang S, Guy ME (2006). GenXers versus boomers: Work motivators and management implications. Public Perform Manag Rev.

